# Supplementary figures and images for: Molecular basis of interactions between CaMKII and α-actinin-2 that underlie dendritic spine enlargement
Source: eLife. 2023 Jul 25;12:e85008. doi: 10.7554/eLife.85008 (PMC10484527; doi:10.7554/eLife.85008)

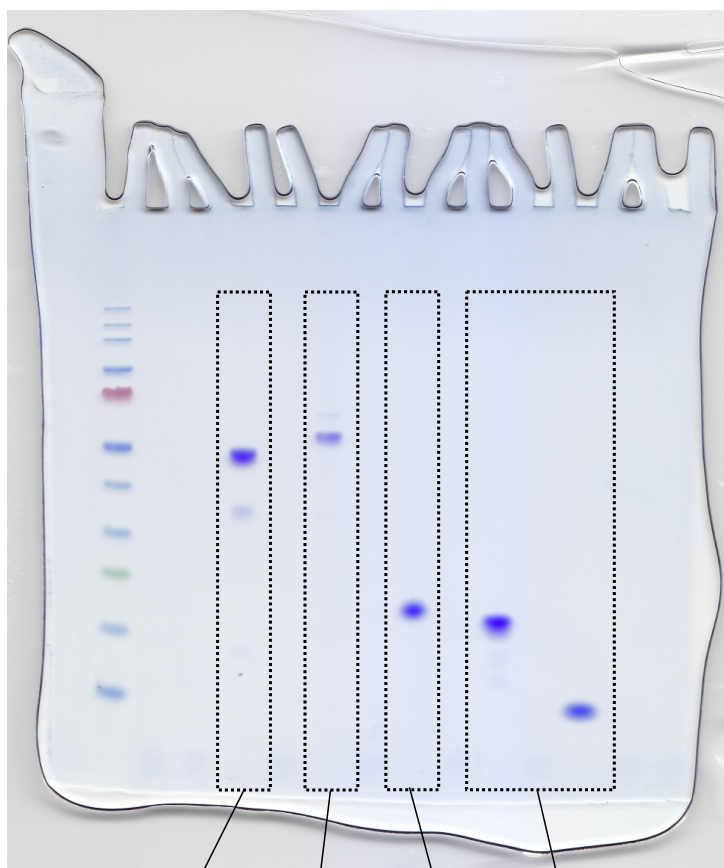

Panel: B D C A

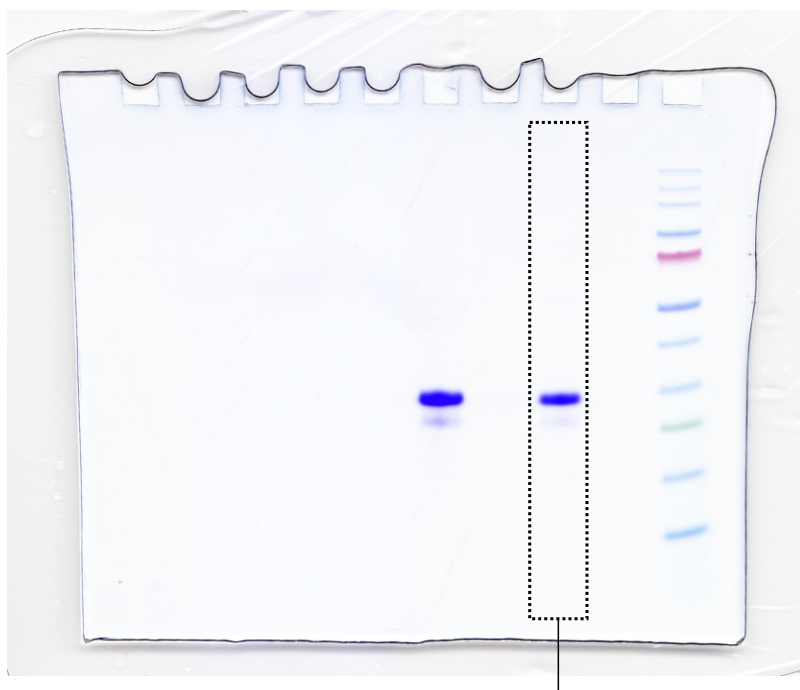

Panel: E

Supplement: Figure 4—figure supplement 1—source data 1. [file elife-85008-fig4-figsupp1-data1.pdf]

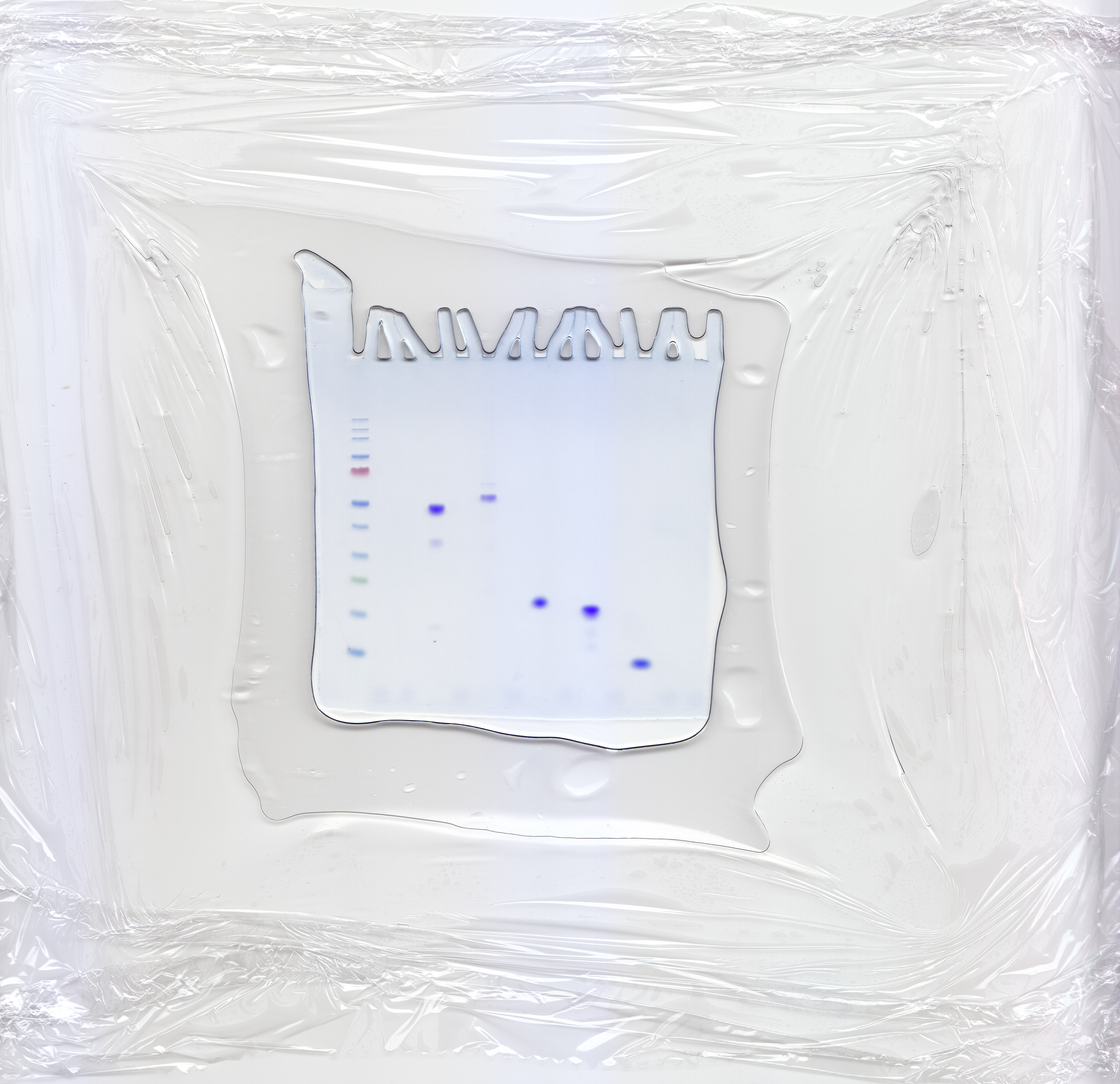

Supplement: Figure 4—figure supplement 1—source data 2. [file elife-85008-fig4-figsupp1-data2.jpg]

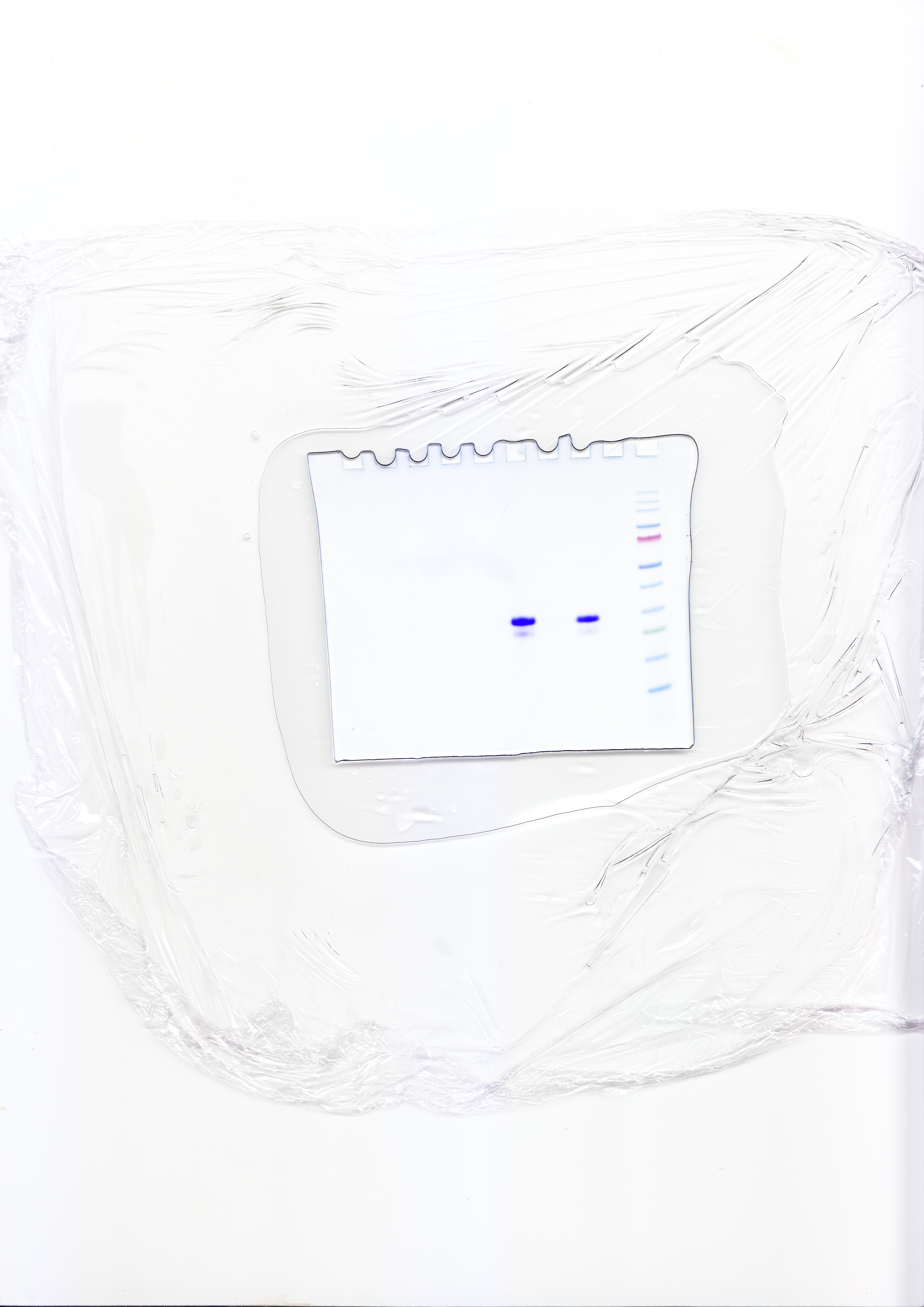

Supplement: Figure 4—figure supplement 1—source data 3. [file elife-85008-fig4-figsupp1-data3.tif]

**D** *Upper sub-panel*

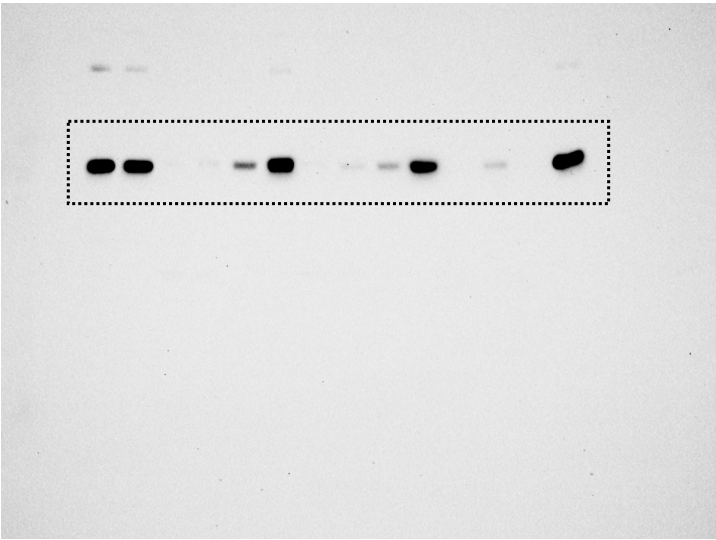

IB: CaMKII $\alpha$

**D** *Lower sub-panel*

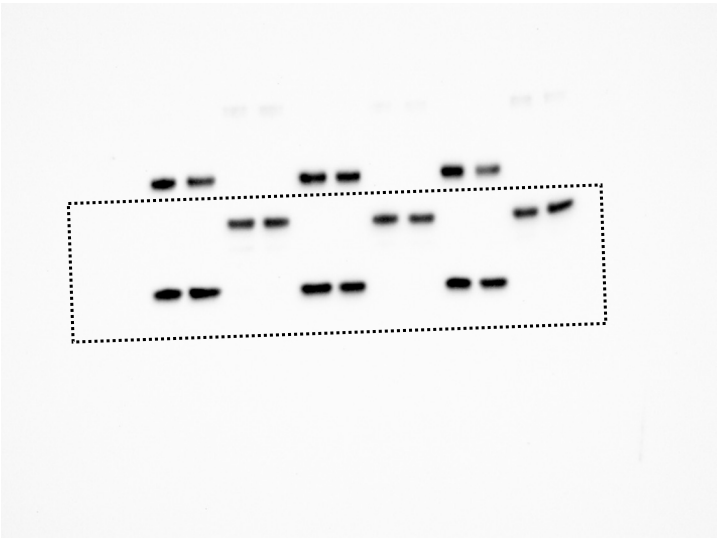

IB: GST

Supplement: Figure 6—source data 2. [file elife-85008-fig6-data2.zip › Actinin_Fig6_SourceData2_UncroppedBlots.pdf]
