# Supplementary material for: Molecular basis of interactions between CaMKII and α-actinin-2 that underlie dendritic spine enlargement
Source: eLife. 2023 Jul 25;12:e85008. doi: 10.7554/eLife.85008 (PMC10484527; doi:10.7554/eLife.85008)
Supplement: Supplementary file 1. [file elife-85008-supp1.docx]

**Supplementary File 1. Data Collection & Refinement Statistics**

|  | **CaMKII peptide +**  **α-actinin-2 EF3-4** | **CaMKII peptide + α-actinin-2 EF3-4 (native sulfur anomalous scattering)** |
| --- | --- | --- |
| **PDB Code** | 6TS3 |  |
| **Data Collection** | | |
| **X-ray wavelength (Å)** | 0.9795 | 2.0664 |
| **Resolution range (Å)** | 44.46-1.28 | 44.52-2.38 |
| **Space group** | P1211 | P1211 |
| **Cell dimensions** |  |  |
| **a, b, c (Å)** | 43.35, 47.27, 47.47 | 43.35, 47.27, 47.47 |
| **α, β, γ (°)** | 90.0, 110.53, 90.0 | 90.0, 110.53, 90.0 |
| **Unique Reflections** | 46303 (2152) | 7653 (521) |
| **Multiplicity** | 6.6 (5.2) | 18.1 (11.8) |
| **Completeness (%)** | 99.8 (95.4) | 94.5 (66.8) |
| **Mean I/σ(I)** | 19.7 (2.4) | 52.2 (26.8) |
| **R_pim_** | 0.015 (0.208) | 0.011 (0.019) |
| **CC_1/2_** | 0.999 (0.94) | 0.999 (0.999) |
| **Anomalous completeness (%)** |  | 92.1 (57.4) |
| **Anomalous multiplicity** |  | 9 (6.4) |
| **Copies per ASU** | 2 | 2 |
| **Refinement statistics** | | |
| **R_work_** | 0.183 |  |
| **R_free_** | 0.199 |  |
| **R.m.s. deviations of bond lengths (Å)** | 0.005 |  |
| **R.m.s. deviations of bond angles (Å)** | 0.698 |  |
| **Ramachandran** |  |  |
| **Favored (%)** | 99.4 |  |
| **Allowed (%)** | 0.6 |  |
| **Outliers (%)** | 0 |  |
