# Supplementary material for: Molecular basis of interactions between CaMKII and α-actinin-2 that underlie dendritic spine enlargement
Source: eLife. 2023 Jul 25;12:e85008. doi: 10.7554/eLife.85008 (PMC10484527; doi:10.7554/eLife.85008)
Supplement: Supplementary file 2. — Primer sequences are shown (5’ to 3’). [file elife-85008-supp2.docx]

**Supplementary File 2. Oligonucleotide primers sequences**

| **Primer Name** | **Sequence (5’ to 3’)** |
| --- | --- |
| EcoRI_FLAG_actinin_1 | CTTGAATTCCACCATGGACTATAAGGATGACGATGACAAAATGAACCAGATAGAGCCCGG |
| actinin_894stop_SalI | CTTGTCGACTCACAGATCGCTCTCCCCG |
| actinin_890stop_SalI | CTTGTCGACTCACCCGTAGAGTGCGGAAGAGAA |
| actinin_748stop_SalI | CTTGTCGACTCACTTCGCATCTCTCGTCAGGATCT |
| L854R_F | GAGGAGCTGCGTCGGGAGCGGCCCCCGGATCAGGCCCAG |
| L854R_R | CTGGGCCTGATCCGGGGGCCGCTCCCGACGCAGCTCCTC |
| pNH-Trx CaMKIIα 1to315_F | TACTTCCAATCCATGGCTACCATCACCTGCACC |
| pNH-Trx CaMKIIα 1to315_R | TATCCACCTTTACTGTCATCGGGAGAAGTTCCTGGTGGC |
| EcoRI_actinin_747 | TACTTGAATTCGCGAAGGGCATCACCCAG |
| actinin_Term_Not1 | TAAATCGCGGCCGCTCACAGATCGCTCTCCCC |
| EF34_F | TACTTCCAATCCAATCCAATGCAACCGACACTGCCGAGCAGGTCATC |
| EF34_R | TTATCCACTTCCAATGTTATTATCATCACAGATCGCTCTCCCCG |
| EcoRI_6HisCaMKIIa | CATGAATTCCACCATGCACCACCATCACCACCATATGGCTACCATCACCTGCAC |
| CaMKIIa_Term_XhoI | CACTCGAGTCAATGCGGCAGGACG |
| BamHI_GluN2B_1260 | AGCGGCGGATCCCTGCAGGAACTGGACCAG |
| GluN2B_Term_EcoRI | CCGGGAGCTGCATGTGTCAGAGG |
| T286A_F | GCCTGCATGCACAGACAGGAGGCCGTGGACTGCCTG |
| T286A_R | GAACTTCTTCAGGCAGTCCACGGCCTCCTGTCTGTG |
| T305A_F | GGAAACTGAAGGGAGCCATCCTCGCCACTATGCTGGCCACCAGGAAC |
| T305A_R | GTTCCTGGTGGCCAGCATAGTGGCGAGGATGGCTCCCTTCAGTTTCC |
| T306A_F | GGAAACTGAAGGGAGCCATCCTCACCGCTATGCTGGCCACCAGGAAC |
| T306A_R | GTTCCTGGTGGCCAGCATAGCGGTGAGGATGGCTCCCTTCAGTTTCC |
| BamHI_V5_CaMKII_F | AGCGGCGGATCCCACCATGGGTAAGCCAATCCCAAACCCCTTGCTGGGTCTCGATAGCACAATGGCTACCATCACCTGCACCCGATTCACAGAA |
| CaMKII_Term_SalI_R | AGCAGGTCGACACGCGTCGTACGTCAATGCG |
| AgeI_4gHA4g_F | CCTCGACCGGTCTGGGGGAGGCGGAGGTTACCCATACGATGTTCCAGATTACGCTGGTGGAGGGGGTGGCAACTTCTGCCGCAGCTGTCCTTCC |
| EcoRI_GluN2B_R | CGGCAAGAATTCAGTGAAAGC |
| CaMKIIa_AQ_F | CGGAACAAAGCGCGCCAGCAGCATTCCTACGACACC |
| CaMKIIa_AQ_R | CTGCTGGCGCGCTTTGTTCCGATTCTTCTTCTGAGC |
